# The American Medical Association

**Published:** 1851-10

**Authors:** 


THE AMERICAN MEDICAL ASSOCIATION—DRS. RAMSEY AND ROBERTSON.

We have received from Dr. Ramsey, of Raysville, Ga., a copy of his appeal to the medical profession, and it is due to professional courtesy and justice, that some notice shall be taken of the controversy in which Dr. Ramsey is involved, with certain members of the medical profession.

Those who have read the report of the proceedings of the American Medical Association, of last May, will remember that while Dr. Storer, chairman of the committee on Obstetrics, was reading his report, he was arrested in the reading by a declaration on the part of Dr. F. M. Robertson, that certain statistics taken from a paper, published by Dr. Ramsey, in the Medical Examiner, of last October, were false, and that the author was not a reliable man. The report was recommitted, and on the following day of the session, Dr. Storer announced that he had erased the statistics in question, and placed the report in the hands of the Association.

In the July number of the Medical Examiner, the courteous and able editors of that journal, made the following just and proper remarks:

"We confess that these proceedings struck us as somewhat extraordinary. We have no personal knowledge of either of the gentlemen named, and we write in the dark as to the under-current of feeling which may have prevailed on the occasion. But, forming our judgment from the record, we cannot but dissent from the decision which was made. Whatever the merits of the case, the ordinary forms of justice were disregarded in the condemnation of an unheard, absent person; and there appears to us to have been in every way a want of propriety in the time, the place, and the tribunal, which were selected for the trial of this question of credibility. The affair seems to have been very much a 'snap judgment.'"

These sentiments seem to us to be eminently correct, and if their spirit prevailed more generally among medical men, the profession and the community would be gainers. While we would severely condemn those who endeavor to manufacture a reputation by false reports, we hold that he who charges a professional brother with heinous offences, should be well fortified with testimony before he undertakes to publish his charges. A man who deliberately libels another, who assails him for conduct of which he was not guilty, and who undertakes by falsehood to undermine the professional reputation of a brother physician, when the truths of the case would have been creditable, is an unfit associate for members of the medical profession, and his proper retribution would be degradation from his position among medical men.

We gather from Dr. Ramsey's pamphlet, that Dr. Robertson felt himself justified in arresting the statistics of Dr. Ramsey before the American Medical Association, for the following reasons: Dr. Cooper, of Ga., had incorporated these statistics in a report which he prepared for the State Medical Society of Georgia, and being, in some way satisfied of the falsity of those statistics, Dr. Cooper had requested permission to withdraw those statements, and the society had accorded to his wishes, thereby, apparently, endorsing Dr. Cooper's reformed views. The first intimation that Dr. Ramsey received of the conduct of the Georgia Medical Society was from Dr. Robertson's letter of exculpation, sent to Dr. De Saussure, and by him forwarded to Dr. Ramsey. This letter was written two months after the meeting of the American Medical Association, and, we repeat, gave Dr. Ramsey the first intimation that he had been accused, tried, condemned and punished by the Georgia Medical Society. And this singular conduct on the part of a State Medical Society was unquestionably the main element for the punishment of Dr. Ramsey before the American Medical Society. We can easily understand Dr. Storer's readiness to erase Dr Ramsey's statistics from the report, when he was told that the State Medical Society of Georgia, had rejected them as false.

In addition to this erroneous issue, other positions were taken. Dr. Robertson condemns Dr. Ramsey on various issues, which we shall briefly detail:

1st. Dr. Robertson charges that Dr. Ramsey reported 673 cases of midwifery, which according to Dr. Robertson yields an average of 67 cases per annum. Now, Dr. Ramsey's report claims no such numbers. *His* statistics claimed 47 and a fraction over per annum. This is a very material difference, which was scarcely justified by Dr. Ramsey's loose guess that he had probably attended 600 or more cases in all.

2d. One of the proofs of Dr. Ramsey's falsity was found in the statement, that Dr. R. had been a practitioner only about ten years, and not continuously in one place. But these are not facts: Dr. Ramsey had been in practice at least twelve years when his paper was published, and had been continuous in one locality during the time embraced in his report.

3d. It was alleged against Dr. Ramsey, that his "portion of country was not densely populated, and in which there were the usual number of competitors for public patronage." But Dr. Ramsey fully establishes the fact, that his neighborhood is "thickly settled," and notwithstanding the competition, Dr. Ramsey had a heavy obstetrical practice, and his "competitors" say, that he had no competition in this branch of professional business.

Dr. Ramsey very triumphantly sustains his professional and moral character, by the testimony of *his medical brethren in his immediate vicinity.* They rely on his statistics, and their confidence, they say, is based on personal knowledge.

We shall not enter upon any examination of the personalities between Drs. Robertson and Ramsey, but we felt that justice required of us the statement we have given of Dr. Ramsey's case. He has been seriously injured before the profession of the United States. He was condemned before the American Medical Association, by a species of "snap judgment," as our confrêres of the Medical Examiner express it, and the medical journals spread before their readers the proceedings of the American Medical Association; and for this reason, we think that Dr. Ramsey's defence should go before the same readers.

We hope that Dr. Ramsey will bear with us in our condemnation of the coarse language he employs too frequently. His provocation was great, the treatment he received was well calculated to rouse his ire, but even that should not have made him forget, that a physician is bound to be a gentleman in all circumstances. When a physician descends to the language of billingsgate and St. Giles, he injures himself more than any one else. Dr. Ramsey's remarks about the "sable heart," "pigs and puppies," and "the Georgia Medical Society," do him more harm than those against whom they are directed. We earnestly wish he had permitted some cool friend to revise his manuscript, and erase and correct freely. But despite all his sins of language, we congratulate him on his triumphant defence. B.

